# The Evil Effects of the Nasal Douche

**Published:** 1882-10-20

**Authors:** Carl Seiler

**Affiliations:** Lecturer on Diseases of the Throat at the University of Pennsylvania; Chief of the Throat Dispensary at the University Hospital, etc.


### THE EVIL EFFECTS OF THE NASAL DOUCHE.

[Read before the Philadelphia County Medical Society, June 28, 1882.]

By CARL SEILER, M. D.

Lecturer on Diseases of the Throat at the University of Pennsylvania ; Chief of the Throat Dispensary at the University Hospital, etc.

Several years ago I read a paper before this Society in which I pointed out the proper manner of using the nasal douche so as to avoid some of the ill effects which may be produced by this instrument. Since then I have given the matter close attention, and have found that the nasal douche, both anterior and posterior, is in many cases of nasal disease a most dangerous instrument to place into the hands of the patient. If we examine the current medical literature, we will find a number of cases reported in which diseases of the middle ear, inflammation of the mucous membrane lining the frontal sinus, meningitis, and even death, were caused more or less directly by the nasal douche; and I have myself seen several cases of acute otitis media caused by water from the douche entering the Eustachian tube.

The question then arises, shall the nasal douche be used at all, and, if so, how shall it be used, and in what class of cases? To answer these queries is the object of my communication.

In the first place, let me reiterate what I said in my former communication,*—viz, that the nasal douche is one of the best means of cleansing the nasal cavities, and that it can be used in many cases without fear of evil consequences, if the proper precautions are observed. These precautions are: 1, that the liquid should be of the temperature of the body; 2, that it should be of the same specific gravity as the serum of the blood, a liquid easily obtained by dissolving an even teaspoonful of common table salt in a pint of water; 3, that the bottom of the vessel should not be elevated above the forehead of the patient using it, for if the vessel is held higher the force of the liquid becomes so great as easily to find its way into the cavities contiguous and communicating with the nasal cavities; and, 4, that while the water flows through the nasal cavities the patient should abstain from swallowing, because during the act of deglutition the mouth of the Eustrahian tube is momentarily opened, thus allowing the fluid to enter the middle ear.

More important, however, than the above precautions, is the proper selection of cases. A glance at the mechanical arrangement of the nasal cavities will show us that a stream of water thrown up through one nostril passes into the post-nasal cavity, and, being prevented from passing into the lower pharynx by the soft palate, which is raised, will flow out through the other nostril. If, as is frequently the case in nasal catarrh, the nostrils are more or less obstructed by deviation of the septum, exostosis or *echondrosis* of the septum (if I may be allowed to coin a word to designate the localized thickening of the cartilaginous portion of the septum), or by anterior or posterior hypertrophies of the erectile tissue covering the turbinated bones and by polypi, the easy outflow of the fluid is prevented, it accumulates in the post-nasal cavity, and is forced into the middle ear, the frontal sinus, and even into the antrum, giving rise to inflammation of the mucous membrane lining these cavities. It frequently occurs that the hypertrophies act as valves, allowing the fluid to pass up, but prevent it from flowing out again. This is especially noticeable in cases of posterior hypertrophies, which, being attached to the turbinated bones by a sort of pedicle, are forced by the inflowing current into the post-nasal cavity, thus making room for the liquid to pass in, but are tightly wedged into the nostril by the return current, and prevent any outflow.

In cases where the tissue is not sufficiently hypertrophied to cause an obstruction to the current of liquid from the nasal douche under ordinary conditions, it will swell up and cause obstruction when an acute congestion is present, or if the fluid used is too cold or not of the proper density. The same objections hold good when the post-nasal syringe or douche is used, for an obstruction in the nostrils also causes in this case an accumulation of liquid in the post-nasal cavity.

It will therefore be seen that the nasal douche should be used only in those cases of nasal disease in which there is no obstruction in the nostrils, but where there is an accumulation of secretion

*Transactions of the Philadelphia County Medical Society, 1879.

which, becoming inspissated, gives rise to the fetid odor noticed in ozæna. A copious stream, such as can only be obtained from the anterior or posterior nasal douche, is needed to remove the dried crusts and thoroughly cleanse the nasal cavities, and I am in the habit of adding some soda or borax to the solution of salt and water, because I have found that an alkaline solution dissolves and dislodges the crusts more readily than a neutral one. The amount of salt should of course be reduced in proportion to the addition of the alkali —*Philadelphia Med. Times.*

